# Electrocardiogram abnormalities in residents in cold homes: a cross-sectional analysis of the nationwide Smart Wellness Housing survey in Japan

**DOI:** 10.1186/s12199-021-01024-1

**Published:** 2021-10-12

**Authors:** Wataru Umishio, Toshiharu Ikaga, Kazuomi Kario, Yoshihisa Fujino, Masaru Suzuki, Shintaro Ando, Tanji Hoshi, Takesumi Yoshimura, Hiroshi Yoshino, Shuzo Murakami, on behalf of the SWH Survey Group

**Affiliations:** 1 Department of Architecture and Building Engineering, School of Environment and Society, Tokyo Institute of Technology, W8-11, 2-12-1, Ookayama, Meguro-ku, Tokyo, 152-8552 Japan; 2 Department of System Design Engineering, Faculty of Science and Technology, Keio University, Yokohama, Kanagawa Japan; 3 Department of Cardiology, Jichi Medical University School of Medicine, Shimotsuke, Tochigi Japan; 4 Department of Environmental Epidemiology, Institute of Industrial Ecological Sciences, University of Occupational and Environmental Health, Kitakyushu, Fukuoka Japan; 5 Department of Emergency Medicine, Ichikawa General Hospital, Tokyo Dental College, Ichikawa, Chiba Japan; 6 Department of Architecture, Faculty of Environmental Engineering, University of Kitakyushu, Kitakyushu, Fukuoka Japan; 7 Tokyo Metropolitan University, Hachioji, Tokyo, Japan; 8 University of Occupational and Environmental Health, Kitakyushu, Fukuoka Japan; 9 Tohoku University, Sendai, Miyagi Japan; 10 Institute for Building Environment and Energy Conservation, Kojimachi, Chiyoda-ku, Tokyo, Japan

**Keywords:** Electrocardiogram, Cardiovascular disease, Indoor temperature, Housing, Winter

## Abstract

**Background:**

Excess winter mortality caused by cardiovascular disease is particularly profound in cold houses. Consistent with this, accumulating evidence indicates that low indoor temperatures at home increase blood pressure. However, it remains unclear whether low indoor temperatures affect other cardiovascular biomarkers. In its latest list of priority medical devices for management of cardiovascular diseases, the World Health Organization (WHO) included electrocardiography systems as capital medical devices. We therefore examined the association between indoor temperature and electrocardiogram findings.

**Methods:**

We collected electrocardiogram data from 1480 participants during health checkups. We also measured the indoor temperature in the living room and bedroom for 2 weeks in winter, and divided participants into those living in warm houses (average exposure temperature ≥ 18 °C), slightly cold houses (12–18 °C), and cold houses (< 12 °C) in accordance with guidelines issued by the WHO and United Kingdom. The association between indoor temperature (warm vs. slightly cold vs. cold houses) and electrocardiogram findings was analyzed using multivariate logistic regression models, with adjustment for confounders such as demographics (e.g., age, sex, body mass index, household income), lifestyle (e.g., eating habit, exercise, smoking, alcohol drinking), and region.

**Results:**

The average temperature at home was 14.7 °C, and 238, 924, and 318 participants lived in warm, slightly cold, and cold houses, respectively. Electrocardiogram abnormalities were observed in 17.6%, 25.4%, and 30.2% of participants living in warm, slightly cold, and cold houses, respectively (*p* = 0.003, chi-squared test). Compared to participants living in warm houses, the odds ratio of having electrocardiogram abnormalities was 1.79 (95% confidence interval: 1.14–2.81, *p* = 0.011) for those living in slightly cold houses and 2.18 (95% confidence interval: 1.27–3.75, *p* = 0.005) for those living in cold houses.

**Conclusions:**

In addition to blood pressure, living in cold houses may have adverse effects on electrocardiogram. Conversely, keeping the indoor thermal environment within an appropriate range through a combination of living in highly thermal insulated houses and appropriate use of heating devices may contribute to good cardiovascular health.

**Trial registration:**

The trial was retrospectively registered on 27 Dec 2017 to the University hospital Medical Information Network Clinical Trials Registry (UMIN-CTR, https://www.umin.ac.jp/ctr/, registration identifier number UMIN000030601).

**Supplementary Information:**

The online version contains supplementary material available at 10.1186/s12199-021-01024-1.

## Background

The World Health Organization (WHO) has issued several guidelines on the prevention of cardiovascular disease (CVD), the world’s leading cause of death [1–5]. These guidelines highlight four important risk factors for CVD: (1) unhealthy diet, (2) physical inactivity, (3) tobacco use, and (4) harmful use of alcohol. To put the guidelines into action, in 2016, WHO and the United States Centers for Disease Control and Prevention (US CDC) launched the Global Hearts Initiative to promote better lifestyle habits, such as reducing salt intake through SHAKE packages [6], increasing physical activity through ACTIVE packages [7], and improving smoking habit through MPOWER packages [8]. However, the ability to improve lifestyle habits is limited because it depends on the efforts of the individual. Meanwhile, improving one’s living environment is attracting attention as an additional approach for preventing CVD.

In the relationship between living environment and CVD, excess winter mortality (EWM), a phenomenon in which the mortality rate rises sharply in winter [9–12], is an inevitable issue. According to estimations by the WHO Regional Office for Europe, 50–70% of EWM is attributed to CVD [13], and EWM caused by CVD is particularly profound in cold houses [14]. The WHO’s 2018 Housing and health guidelines [15] list “low indoor temperatures and insulation” as one of five priority issues. The guidelines indicate that the mechanism of cardiovascular (CV) events is partially explained by a rise in blood pressure (BP) due to cold exposure. Consistent with this, studies finding a relationship between indoor temperature and BP are accumulating [16–18]. However, it remains unclear whether low indoor temperatures affect other CV biomarkers.

Electrocardiogram (ECG), a test that measures the heart’s electrical activity, is one of the most common methods used to assess CV health. In their latest list of priority medical devices for management of CVD, the WHO included ECG as a capital medical device for early detection of CVD [19]. Additionally, previous studies have reported close associations between ECG findings and CVD risk [20, 21]. Thus, it would be valuable to verify whether low indoor temperatures are associated with ECG abnormalities. However, the association has not been well investigated.

We conducted a nationwide epidemiological survey on housing and health in Japan, named the Smart Wellness Housing (SWH) survey. In Japan, an estimated 39% of existing houses are uninsulated [22], and a large proportion of residents live in houses with low indoor temperatures [23]. There is a concern that living in such houses may have adverse effects on health. The aim of this paper was to determine the association between the indoor temperature at home and ECG findings.

## Methods

### Ethical issues

The study was conducted according to the principles of the Declaration of Helsinki. The study protocol and informed consent procedure were approved by the Hattori Clinic Ethics Review Board (Approval No. S1410-J03). This review board consists of experts in medicine, bioethics, and law and is certified by the Ministry of Health, Labour, and Welfare (Accreditation No. CRB3180027). The study protocol was registered at the University hospital Medical Information Network Clinical Trials Registry (UMIN000030601). All participants provided written informed consent to participate and to have their data published as a group.

### Study design

The aims and study design of the SWH survey are reported elsewhere [18]. Briefly, the survey was conducted as a non-randomized controlled trial with an insulation retrofitting group and non-insulation retrofitting (control) group to examine the health benefits of insulation retrofitting houses. Participants were recruited by construction companies throughout all 47 prefectures in 8 regions in Japan (Figure S1). Inclusion criteria were (1) intention to conduct insulation retrofitting, (2) age over 20 years, and (3) pre-renovation house that did not meet S (Supreme) standards of the “Act on the Promotion of Dissemination of Long-Lasting Quality Housing” in Japan. In this paper, we performed a cross-sectional analysis of data obtained in the baseline (before insulation retrofitting) survey of FY 2014 to 2017. We focused on data obtained before insulation retrofitting to reflect the actual condition of houses in Japan, most of which have low insulation performance [22].

### Electrocardiogram measurements

Participants were asked to submit the results of a health checkup which was conducted within a year of the survey period. In Japan, health checkups conducted by doctors are required once a year in accordance with the Industrial Safety and Health Act. Items included in the health checkup are (1) past medical history; (2) subjective and objective symptoms; (3) height, weight, abdominal girth, and visual and hearing acuity; (4) chest X-ray; (5) BP; (6) anemia; (7) liver function; (8) blood lipids; (9) blood glucose; (10) urine; and (11) ECG. We examined ECG data in the present study. The 12-lead ECG test requires that participants lay on their back on a bed while a medical technologist places electrodes on their chest, wrists, and ankles to obtain ECG waveforms over a period of a few minutes. Doctors judged whether or not participants had abnormal ECG waveforms. Examples of abnormalities in ECG are shown in Table S1.

### Indoor temperature measurements

Indoor temperature and relative humidity at 1.0 m above the floor were measured in participants’ living room and bedroom at 10-min intervals (TR-72wf; T&D Corp., Nagano, Japan) in winter (November–March). When installing the temperature and humidity loggers, the device was placed (1) out of direct sunlight and (2) far away from heating equipment or heat-generating devices like refrigerators and televisions to avoid extreme outlier measurements. Outdoor temperature was obtained from the closest local meteorological observatory to each participant’s house.

### Other measurements

Participants were also asked to complete a questionnaire survey that inquired about demographics such as age, sex, height, weight, and household income; lifestyle indicators such as eating habit, exercise, smoking, and alcohol consumption; and health conditions related to CVD. Participants indicated their household income by choosing from multiple choices, which were later reclassified as low (< 2 million JPY), middle (2–6 million JPY), and high (≥ 6 million JPY) in accordance with the National Health and Nutrition Survey. The salt check sheet score and frequency of vegetable intake were used as measures of eating habit, with the former classified in 4 groups (low (0–8 points), medium (9–13 points), high (14–19 points), or very high (≥ 20 points)) [24] and the latter classified according to whether or not participants ate vegetables regularly. Exercise, smoking, and alcohol drinking were evaluated as two-valued variables; that is, whether or not participants did regular moderate exercise, whether or not they were a current smoker, and whether or not they were a current drinker, respectively. Regarding health conditions, participants were asked whether they visited the hospital for a list of diseases. A diary survey was also conducted, in which participants provided details of their waking time, bedtime, and time spent at home on a daily basis.

### Statistical analysis

First, we defined warm, slightly cold, and cold houses. We calculated participants’ exposure temperature by extracting the living room temperature when participants were at home (excluding the period while they were asleep) and the bedroom temperature while they were asleep based on information from the diary survey. Details of the definition of participants’ exposure temperature are shown in Figure S2. Subsequently, we divided participants into three groups according to the average exposure temperature at home: ≥ 18 °C (warm houses), 12–18 °C (slightly cold houses), and < 12 °C (cold houses). These thresholds were chosen in accordance with the WHO Housing and health guidelines for “a safe and well-balanced indoor temperature” during cold seasons (18 °C) [15] and a UK report on the temperature at which CVD risk begins to increase (12 °C) [25].

Inter-group comparisons of proportion values were performed using the chi-squared test. The association between exposure temperature at home and ECG findings was evaluated using univariate and multivariate logistic regression analyses. Whether or not participants’ ECG findings were abnormal was inputted as the objective variable, and the exposure temperature (warm vs. slightly cold vs cold houses) was inputted as the explanatory variable. The analyses were adjusted for participants’ basic characteristics such as age (≥ 65 or < 65 years), sex, body mass index (BMI = weight[kg]/height[m]^2^; ≥ 25 or < 25 kg/m^2^), household income (low, middle, or high), salt check sheet score (low, medium, high, or very high), vegetable intake (regularly or not), exercise habit (regularly or not), smoking status (currently or not), alcohol drinking status (currently or not), antihypertensive drug use (currently or not), outdoor temperature in winter (as a continuous variable), and region in Japan (8 regions in Figure S1 as a dummy variable). Additionally, the season during which the health checkup was performed (whether or not it was in winter) was inputted into the model to account for seasonal variations. All *P* values were two sided, and a two-sided *P* value less than 0.05 was considered statistically significant. All analyses were performed using SPSS Ver. 26 (SPSS Inc., Chicago, Illinois, USA).

## Results

### Definition of warm, slightly cold, and cold houses based on indoor temperature

**Fig. 1:**
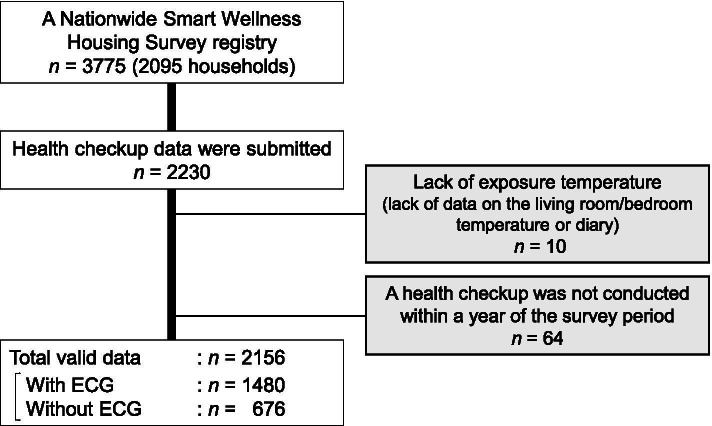
Flow of the selection of valid samples*. ECG* electrocardiogram

Figure 1 shows the selection of valid samples. Of 3775 participants in the SWH survey, 2230 submitted health checkup data and 2156 had valid data. Valid samples had different health checkup items because the items can be omitted at the doctors’ discretion. A total of 1480 participants had ECG data.

**Fig. 2:**
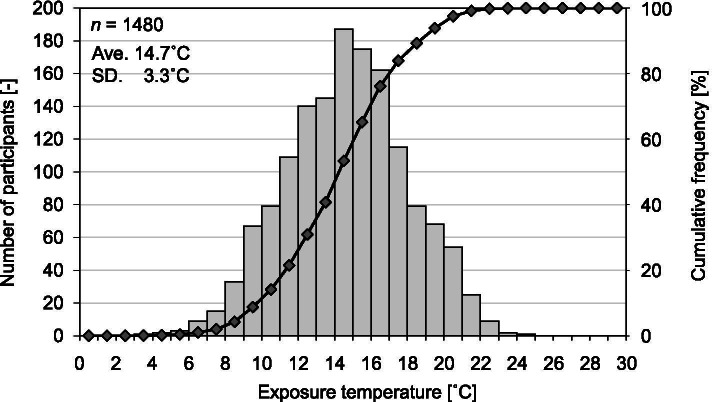
Distribution of average exposure temperature

**Fig. 3:**
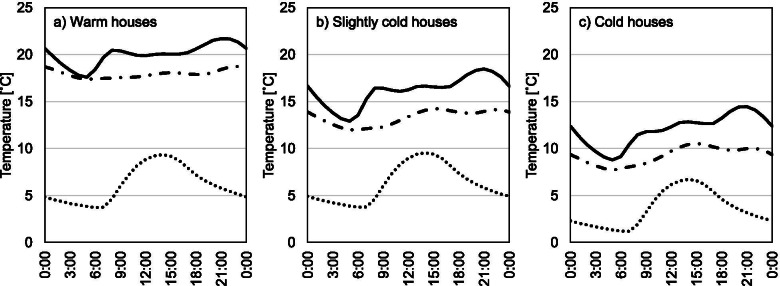
Fluctuation of indoor and outdoor temperature throughout a day in the three groups. Solid line shows the living room temperature, dash-dot-dash line shows the bedroom temperature, and dotted line shows the outdoor temperature

The distribution of exposure temperature is shown in Fig. 2. (The distribution of average exposure time to living room and bedroom temperature is also shown in Figure S3.) The average exposure temperature was 14.7 °C. Based on the results of Fig. 2, we divided the participants into those living in warm (≥ 18 °C), slightly cold (12–18 °C), and cold (< 12 °C) houses. The sample size of the three groups was 238, 924, and 318, respectively. Figure 3 shows the changes in indoor and outdoor temperature throughout a day in the three groups. The average living room/bedroom temperature was 20.0/18.0 °C for warm houses, 16.2/13.3 °C for slightly cold houses, and 12.0/9.3 °C for cold houses. Although the outdoor temperature of warm houses was almost the same as that of slightly cold houses, the average living room/bedroom temperature differed by 3.9/4.7 °C. Focusing on intra-day variations, all three groups showed the same trend in which the living room and bedroom temperature dropped to the minimum value at 5 AM. Subsequently, only the living room temperature rose sharply in the morning and gradually increased to the maximum value at 9 PM.

### Study profile

Table 1 shows the baseline characteristics of the participants overall and by group. Regarding locations, participants living in the Kanto, Chubu, and Kinki region, which are populated areas in Japan, accounted for 60%. In the Hokkaido region where outdoor temperature is the lowest in Japan, there were many participants living in warm houses. On the other hand, in the Kyushu region which is located in the southwestern area of Japan and considered to have a mild climate, a large number of participants lived in slightly cold or cold houses. The above results suggested the necessity to consider regions in the following analyses.

Regarding individual attributes, about half of the participants were men, the average age was 56 years and about 25% were 65 years and older. The average BMI was 22.8 kg/m2 and approximately 20% were classified as overweight. Further, 45% of participants were from high income households, which is a larger proportion than that reported by the National Health and Nutrition Survey in Japan (27.4%). While the number of patients with stroke, angina/myocardial infarction, and diabetes was small, more than 10% had hyperlipidemia and hypertension. Participants’ basic characteristics were adjusted in the subsequent multivariate logistic regression model to account for differences among the three groups.

**Table 1:** Basic characteristics of participants in the baseline survey in winter

Variable	Overall	Warm (≥ 18 °C)	Slightly cold (12–18 °C)	Cold (< 12 °C)	p for the *χ*^2^ test
	*N* (%)	*N* (%)	*N* (%)	*N* (%)	–
Location					
Region					< 0.001
Hokkaido region	73 (5)	37 (16)	32 (3)	4 (1)	
Tohoku region	156 (11)	15 (6)	78 (8)	63 (20)	
Kanto region	280 (19)	60 (25)	173 (19)	47 (15)	
Chubu region	305 (21)	38 (16)	200 (22)	67 (21)	
Kinki region	297 (20)	48 (20)	194 (21)	55 (17)	
Chugoku region	98 (7)	14 (6)	58 (6)	26 (8)	
Shikoku region	69 (5)	14 (6)	44 (5)	11 (3)	
Kyushu region	202 (14)	12 (5)	145 (16)	45 (14)	
Demographics					
Age (≥ 65 years)	352 (24)	44 (18)	222 (24)	86 (27)	0.061
Men	816	136 (57)	518 (56)	162 (51)	0.227
Body mass index (≥ 25 kg/m^2^)	318 (21)	53	197 (21)	68 (21)	0.950
Household income					0.007
Low (< 2 million JPY)	105 (8)	12 (6)	62 (7)	31 (10)	
Middle (2−6 million JPY)	647 (47)	105 (48)	384 (45)	158 (53)	
High (≥ 6 million JPY)	616 (45)	101 (46)	407 (48)	108 (36)	
Lifestyle					
Salt check sheet					0.617
Low (0−8 points)	180 (13)	28 (12)	109 (12)	43 (14)	
Medium (9−13 points)	566 (40)	101 (44)	357 (41)	108 (36)	
High (14−19 points)	555 (39)	84 (36)	349 (40)	122 (41)	
Very high (≥20 points)	112 (8)	18 (8)	66 (7)	28 (9)	
Regular vegetable intake	1111 (75)	179 (75)	687 (75)	245 (77)	0.716
Regular exercise	441 (30)	66 (28)	255 (28)	120 (38)	0.003
Current smoker	224 (16)	40 (18)	138 (16)	46 (16)	0.803
Current drinker	872 (60)	141 (60)	568 (62)	163 (52)	0.005
Antihypertensive drug use	325 (23)	35 (15)	212 (24)	78 (25)	0.007
Health condition					
Stroke	17 (1)	4 (2)	9 (1)	4 (1)	0.649
Angina/Myocardial infarction	35 (2)	5 (2)	22 (2)	8 (3)	0.944
Diabetes	87 (6)	14 (6)	59 (7)	14 (5)	0.452
Hyperlipidemia	259 (18)	31 (13)	167 (19)	61 (20)	0.107
Hypertension	312 (22)	31 (13)	207 (23)	74 (24)	0.003

The proportion (%) was calculated excluding missing values

### Comparison of electrocardiogram data among warm, slightly cold, and cold houses

**Fig. 4:**
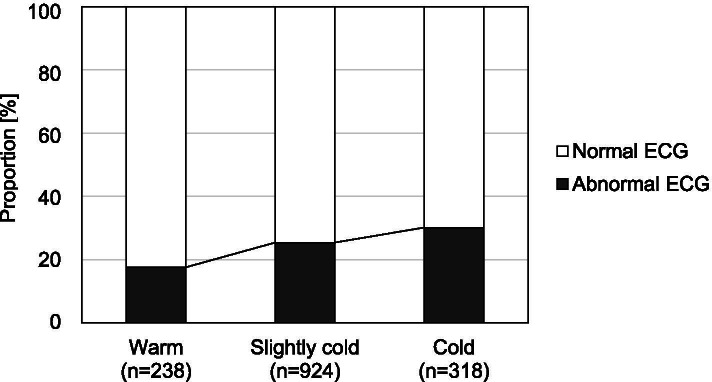
Proportion of residents with electrocardiogram abnormalities in the three groups. *ECG* electrocardiogram

Figure 4 shows the proportion of participants with ECG abnormalities living in warm, slightly cold, and cold houses. The number of participants with ECG abnormalities increased with colder houses. ECG abnormalities were identified in 17.6%, 25.4%, and 30.2% of participants living in warm, slightly cold, and cold houses, respectively (*p* = 0.003, chi-squared test). The results of logistic regression analyses are shown in Table 2. Compared to participants living in warm houses, the adjusted odds ratio of having ECG abnormalities was 1.79 (95%CI: 1.14–2.81, *p* = 0.011) for those living in slightly cold houses and 2.18 (95%CI: 1.27–3.75, *p* = 0.005) for those living in cold houses.

**Table 2:** Associations between electrocardiogram data and indoor temperature (warm vs. slightly cold vs cold houses)

	Unadjusted			Adjusted*		
Explanatory variable	Odds ratio	(95%CI)	*p*	Odds ratio	(95%CI)	*p*
Warm (≥ 18°C)	Ref.			Ref.		
Slightly cold (12–18 °C)	1.83	(1.20, 2.80)	0.005	1.79	(1.14, 2.81)	0.011
Cold (< 12 °C)	2.14	(1.32, 3.45)	0.002	2.18	(1.27, 3.75)	0.005

*Adjusted for region in Japan, age, sex, body mass index, household income, salt check sheet score, vegetable consumption, exercise, smoking, alcohol drinking, antihypertensive drug use, outdoor temperature in winter, and season of health checkup

## Discussion

To our knowledge, this is the first study to assess the association between ECG abnormalities and daily exposure temperature measured in participants’ homes across 2 weeks in winter. Although a large number of studies have examined seasonal variations in CV biomarkers such as blood pressure [26–29], blood lipids [30–32], and blood glucose level [33, 34] and the relationship between these biomarkers and outdoor temperature [35–38], few studies have examined the relationship between CV biomarkers and the indoor temperature at home. Unlike the outdoor temperature, the indoor temperature is a controllable factor. Therefore, evidence on the relationship between CV biomarkers and indoor temperature can be used to prevent CVD, and in turn reduce EWM.

As mentioned previously, there is accumulating evidence that low indoor temperatures increase BP [16–18]. However, because BP changes from beat to beat, it is unclear whether the effect of low indoor temperatures on CV health is transient. Therefore, it is important to clarify the association between indoor temperature and other CV biomarkers. Shiue [39] analyzed the relationship between indoor temperature and CV biomarkers using data obtained from nurses’ interviews of 7997 participants and showed that those living in houses with indoor temperatures below 18 °C had poor biomarker values. Saeki et al. [40] found a significant and independent association between low indoor temperatures and high platelet counts among 1095 elderly participants. However, these studies did not assess ECG, which reflects CV health and is recommended by WHO as an early detection and preventive method for CVD [19]. Thus, we expect our finding that low indoor temperatures are linked to ECG abnormalities will contribute to the progress of studies on housing and health.

A potential mechanism underlying the association between ECG abnormalities and low indoor temperatures is that daily cold stress stimulates sympathetic activity, which can lead to arrhythmias or cause coronary spasms to result in myocardial ischemia. Further, a large body of research has shown that hypertension causes ECG abnormalities such as left ventricular hypertrophy [41], myocardial infarction [42], arrhythmias [43], and atrioventricular block [44]. Based on evidence that low indoor temperatures increase BP [16–18], hypertension caused by living in cold houses may result in ECG abnormalities. Thus, rather than having only transient effects on blood pressure, living in cold houses may in fact have cumulative effects on ECG. These findings strengthen the significance of living in warm houses for the prevention of CV events.

In conjunction with lifestyle modifications, to reduce future CVD risk, we recommend improving the indoor home thermal environment. Previous systematic reviews have shown that interventions on lifestyle habits in long-term studies [45] or community studies [46] do not effectively reduce CVD risk. It may therefore be more effective to improve one’s living environment simultaneously. There are 2 main strategies for improving the home thermal environment: live in a highly thermal insulated house and use heating devices. As shown in Fig. 3, while the heating pattern (the fluctuation in indoor temperature) and outdoor temperature were comparable between warm and slightly cold houses, the indoor temperature level was markedly different. This difference was driven in part by differences in thermal insulation levels. Furthermore, bedroom temperatures in the three groups were lower than living room temperatures throughout the day, which may be because partial heating of only the living room has become a habit in Japan. There is clearly room for improvement in the strategies used to increase the exposure temperature at home. Both strategies for improving the home thermal environment have strengths: living in a highly insulated house improves the thermal environment at an unconscious level, and using heating devices is a more practical choice in terms of time and cost. Thus, the combination of the two is recommended to improve the home thermal environment.

A major strength of the present study was that we used objective ECG data and 2-week indoor temperature measurements, which may have reduced biases due to the interposition of consciousness. Nevertheless, this study had several limitations. First, there is a selection bias because health checkup items were omitted at the doctors’ discretion. So, valid samples might be biased toward participants at high risk of CVD. In fact, there are differences in basic characteristics between participants with ECG (*n* = 1480) and without ECG data (*n* = 676) (Table S2). Second, the time of the year during which participants conducted their health checkup varied from person to person. However, we inputted the season in which the health checkup was conducted (whether or not it was in winter) into the logistic model to adjust for seasonal variations in the CV biomarker. Third, we could not examine the association between indoor temperature and specific ECG abnormalities (e.g., arrhythmia, myocardial infarction, and atrial fibrillation) because of the small sample size. Finally, ECG monitoring was conducted for a relatively short time during the health checkup. In contrast to standard ECG, ambulatory ECG provides more information on an individual’s heart health during daily life [47, 48]. We, therefore, suggest that future research should include a subgroup in which ambulatory ECG monitoring is conducted and evaluate the relationship between ECG and indoor temperature based on long-term observations.

## Conclusions

The present study showed a significant association between cold indoor temperatures and ECG abnormalities based on a cross-sectional study of 1480 participants in real-world settings. Our findings provide new insight in the field of housing and health. A clinical implication of our study is that keeping the indoor thermal environment within an appropriate range may contribute to good cardiovascular health. Besides lifestyle modifications, living in houses with high thermal insulation and appropriate use of heating devices can reduce CVD, and in turn EWM. Future long-term follow-up studies that compare residents in warm houses with those in cold houses should be conducted to confirm our findings.

## Supplementary Information

**Additional file 1: Table S1.** Details of abnormalities in electrocardiogram. **Table S2.** Basic characteristics of participants with and without ECG data. **Table S3.** Members of Smart Wellness Housing Survey Group. **Figure S1.** 47 prefectures in 8 regions in Japan. **Figure S2.** Definition of participants’ exposure temperature. **Figure S3.** Distribution of average exposure time to living room and bedroom temperature.

## Data Availability

The data presented in this study are available on request from the corresponding author. The data are not publicly available due to ethical restrictions.

## Abbreviations

BMI: Body mass index
BP: Blood pressure
CV: Cardiovascular
CVD: Cardiovascular disease
ECG: Electrocardiogram
EWM: Excess winter mortality
JPY: Japanese Yen
SWH: Smart Wellness Housing
UK: United Kingdom
US CDC: United States Centers for Disease Control and Prevention
WHO: World Health Organization
