# Supplementary material for: Electrocardiogram abnormalities in residents in cold homes: a cross-sectional analysis of the nationwide Smart Wellness Housing survey in Japan
Source: Environ Health Prev Med. 2021 Oct 12;26:104. doi: 10.1186/s12199-021-01024-1 (PMC8513347; doi:10.1186/s12199-021-01024-1)
Supplement: Supplementary file 1 — Additional file 1: Table S1. Details of abnormalities in electrocardiogram. Table S2. Basic characteristics of participants with and without ECG data. Table S3. Members of Smart Wellness Housing Survey Group. Figure S1. 47 prefectures in 8 regions in Japan. Figure S2. Definition of participants’ exposure temperature. Figure S3. Distribution of average exposure time to living room and bedroom temperature. [file 12199_2021_1024_MOESM1_ESM.docx]

*Online Supplement*

Electrocardiogram abnormalities in residents in cold homes:

A cross-sectional analysis of the nationwide Smart Wellness Housing survey in Japan

Wataru Umishio^1,2,*^, Toshiharu Ikaga^2^, Kazuomi Kario^3^, Yoshihisa Fujino^4^, Masaru Suzuki^5^, Shintaro Ando^6^, Tanji Hoshi^7^, Takesumi Yoshimura^8^, Hiroshi Yoshino^9^, Shuzo Murakami^10^, on behalf of the SWH survey group

1 Department of Architecture and Building Engineering, School of Environment and Society, Tokyo Institute of Technology, Meguro, Tokyo, Japan

2 Department of System Design Engineering, Faculty of Science and Technology, Keio University, Yokohama, Kanagawa, Japan

3 Department of Cardiology, Jichi Medical University School of Medicine, Shimotsuke, Tochigi, Japan

4 Department of Environmental Epidemiology, Institute of Industrial Ecological Sciences, University of Occupational and Environmental Health, Kitakyushu, Fukuoka, Japan

5 Department of Emergency Medicine, Ichikawa General Hospital, Tokyo Dental College, Ichikawa, Chiba, Japan

6 Department of Architecture, Faculty of Environmental Engineering, The University of Kitakyushu, Kitakyushu, Fukuoka, Japan

7 Tokyo Metropolitan University, Hachioji, Tokyo, Japan

8 University of Occupational and Environmental Health, Kitakyushu, Fukuoka, Japan

9 Tohoku University, Sendai, Miyagi, Japan

10 Institute for Building Environment and Energy Conservation, Chiyoda, Tokyo, Japan

* Correspondence: umishio.w.aa@m.titech.ac.jp; Tel.: +81-3-5734-2908

Table S1. Details of abnormalities in electrocardiogram

| Category | Diagnostic statement |
| --- | --- |
| Sinus node rhythms and arrhythmias | Sinus tachycardia  Sinus bradycardia  Sinus arrhythmia |
| Supraventricular arrhythmias | Atrial premature complex(es)  Atrial rhythm  Junctional rhythm  Supraventricular complex(es) |
| Supraventricular tachyarrhythmias | Atrial fibrillation  Supraventricular tachycardia |
| Ventricular arrhythmias | Ventricular premature complex(es) |
| Ventricular tachyarrhythmias | Wide-QRS tachycardia |
| Atrioventricular conduction | Short PR (PQ) interval  Prolonged PR (PQ) interval  Atrioventricular conduction disturbance  First-degree atrioventricular block  Atrioventricular dissociation |
| Intraventricular and intra-atrial conduction | Left anterior fascicular block  Left posterior fascicular block  Left bundle-branch block  Intraventricular conduction delay  Incomplete right bundle-branch block  Right bundle-branch block |
| Axis and voltage | Right-axis deviation  Left-axis deviation  Indeterminate axis  High voltage  Low voltage |
| Chamber hypertrophy or enlargement | Left ventricular hypertrophy  Right ventricular hypertrophy |
| ST segment, T wave, and U wave | ST deviation  ST deviation with T-wave change  T-wave abnormality  Prolonged QT interval  Early repolarization |
| Myocardial infarction | Anterior myocardial infarction  Inferior myocardial infarction  Anteroseptal myocardial infarction  Old myocardial infarction |
| Pacemaker | Pacemaker rhythm |

Table S1. Details of abnormalities in electrocardiogram (continued)

| Category | Diagnostic statement |
| --- | --- |
| Others | Clockwise rotation  Counterclockwise rotation  Q-wave abnormality  Left atrial load  Poor R Wave Progression  RSR' pattern  Right anterior oblique view  Acute right ventricular strain  Myocardial damage/injury  Brugada abnormality  Myocardial ischemia |

Table S2. Basic characteristics of participants with and without ECG data

| Variable | Participants  with ECG data | |  | Participants  without ECG data | | *p* for the  χ^2^ test |
| --- | --- | --- | --- | --- | --- | --- |
|  | N | (%) |  | N | (%) | - |
| Location |  |  |  |  |  |  |
| Region  Hokkaido region  Tohoku region  Kanto region  Chubu region  Kinki region  Chugoku region  Shikoku region  Kyushu region | 73  156  280  305  297  98  69  202 | (5)  (11)  (19)  (21)  (20)  (7)  (5)  (14) |  | 43  43  91  135  134  48  54  128 | (6)  (6)  (13)  (20)  (20)  (7)  (8)  (19) | <0.001 |
| Demographics |  |  |  |  |  |  |
| Age (≥65 years) | 352 | (24) |  | 321 | (47) | <0.001 |
| Men | 816 | (55) |  | 289 | (43) | <0.001 |
| Body mass index (≥25 kg/m^2^) | 318 | (21) |  | 134 | (20) | 0.410 |
| Household income  　Low (<2 million JPY)  Middle (2−6 million JPY)  High (≥6 million JPY) | 105  647  616 | (8)  (47)  (45) |  | 88  351  181 | (14)  (57)  (29) | <0.001 |
| Lifestyle |  |  |  |  |  |  |
| Salt check sheet  Low (0−8 points)  Medium (9−13 points)  High (14−19 points)  Very high (≥20 points) | 180  566  555  112 | (13)  (40)  (39)  (8) |  | 95  267  231  40 | (15)  (42)  (36)  (6) | 0.207 |
| Regular vegetable intake | 1111 | (75) |  | 544 | (81) | 0.002 |
| Regular exercise | 441 | (30) |  | 247 | (37) | 0.001 |
| Current smoker | 224 | (16) |  | 77 | (13) | 0.058 |
| Current drinker | 872 | (60) |  | 309 | (46) | <0.001 |
| Antihypertensive drug use | 325 | (23) |  | 199 | (31) | <0.001 |
| Health condition |  |  |  |  |  |  |
| Stroke | 17 | (1) |  | 17 | (3) | 0.027 |
| Angina/Myocardial infarction | 35 | (2) |  | 31 | (5) | 0.007 |
| Diabetes | 87 | (6) |  | 67 | (10) | 0.001 |
| Hyperlipidemia | 259 | (18) |  | 149 | (23) | 0.008 |
| Hypertension | 312 | (22) |  | 192 | (30) | <0.001 |

The proportion (%) was calculated excluding missing values.

Table S3. Members of Smart Wellness Housing Survey Group

(a) Members of the Research Committee for the Promotion of Smart Wellness Housing

| **Chairperson** | |
| --- | --- |
| Shuzo MURAKAMI * | Institute for Building Environment and Energy Conservation |
| **Vice-chairperson** | |
| Takesumi YOSHIMURA * | University of Occupational and Environmental Health |
| Hiroshi YOSHINO * | Tohoku University |
| Kazuomi KARIO * | Jichi Medical University |
| **Organizer** | |
| Toshiharu IKAGA * | Keio University |
| **Committee member in medicine** | |
| Suminori AKIBA | Kagoshima University |
| Mikio ARITA | Sumiya Rehabilitation Hospital |
| Michiya IGASE | Ehime University |
| Masayoshi ICHIBA | Saga University |
| Nami IMAI | Mie University |
| Masaki UEMURA | At Home, LLC |
| Hiroyuki UEHARA | National Assembly Promoting Healthy and Energy Conserving Housing |
| Haruo UGUISU | Tokushima Bunri University |
| Kensuke ESATO | Yamaguchi University |
| Akira EBOSHIDA | Hiroshima University |
| Yuko OGUMA | Keio University |
| Toshiyuki OJIMA | Hamamatsu University School of Medicine |
| Shimato ONO | Marugame Ono Clinic |
| Yoshio OMATA | Hoju, Co., Ltd. |
| Takahiko KATOH | Kumamoto University |
| Masahiko KATO | Tottori University |
| Shinya KUNO | University of Tsukuba |
| Kiyokage KUBO | Kubo Clinic |
| Yoshiki KURODA | University of Miyazaki |
| Yasuaki SAIJO | Asahikawa Medical University |
| Kazuhiro SATO | University of Fukui |
| Eiji SHIBATA | Yokkaichi Nursing and Medical Care University |
| Kuninori SHIWAKU | Shimane University |
| Narufumi SUGANUMA | Kochi University |

(a) Members of the Research Committee for the Promotion of Smart Wellness Housing (continued)

| **Committee member in medicine (continued)** | |
| --- | --- |
| Tomotaka SOBUE | Osaka University |
| Toshiro TAKEZAKI | Kagoshima University |
| Masatoshi TANAKA | Fukushima Medical University |
| Tsuyoshi TANABE | Yamaguchi University |
| Susumu TSUKAMOTO | Saitama Jikei Hospital |
| Hiroyuki DOI | Okayama University |
| Kunio DOBASHI | Jobu Hospital for Respiratory Diseases |
| Chisato NAGATA | Gifu University |
| Hiroyuki NAKAMURA | Kanazawa University |
| Kunio NAKAYAMA | Former Osaka University |
| Norihiro NOGATA | Saiseikai Karatsu Hospital |
| Takashi HANATO | Eigenji Clinic |
| Yoshihisa FUJINO * | University of Occupational and Environmental Health |
| Tanji HOSHI * | Tokyo Metropolitan University |
| Satoshi HOSHIDE | Jichi Medical University |
| Takahiro MAEDA | Nagasaki University |
| Muneo MINOSHIMA | Minoshima Clinic |
| Takashi MURAWAKA | Yumemokuba, SNPC |
| Hidekazu YAMADA | Kindai University Nara Hospital |
| Misako YOSHINAGA | Kusunoki Hospital |
| **Committee member in architecture** | |
| Akihiko IWASA | Hosei University |
| Atsushi IWAMAE * | Kindai University |
| Akihito OZAKI | Kyushu University |
| Satoru KUNO | Nagoya University |
| Minoru KUMANO | Miyazaki University |
| Shoichi KOJIMA | Saga University |
| Yasuyuki SHIRAISHI | University of Kitakyushu |
| Hirotaka SUZUKI | Hokkaido Research Organization |
| Tsuyoshi SEIKE * | Tokyo University |
| Naoki TAKAGI | Shinshu University |
| Masaki TAJIMA | Kochi University of Technology |

(a) Members of the Research Committee for the Promotion of Smart Wellness Housing (continued)

| **Committee member in architecture (continued)** | |
| --- | --- |
| Yoshito TANAKA | Nagasaki Institute of Applied Science |
| Takayuki TAMAI | National Institute of Technology, Yonago College |
| Mitsutaka TSUJI | Gifu Academy of Forest Science and Culture |
| Reiji TOMIKU | Oita University |
| Hisaya NAGAI | Mie University |
| Daisaku NISHINA | Hiroshima University |
| Hideyo NIMIYA | Kagoshima University |
| Kenichi HASEGAWA | Akita Prefectural University |
| Hirofumi HAYAMA * | Hokkaido University |
| Akira FUKUSHIMA | Former Hokkaido University of Science |
| Yuji HORI | University of Toyama |
| Takeo MATSUOKA | Asia University |
| Teruaki MITAMURA | Maebashi Institute of Technology |
| Shinji YOSHIDA | Nara Women's University |

*: members of the Research Planning Committee for the Promotion of Smart Wellness Housing

(b) Members of the Subcommittee for Analysis of the Smart Wellness Housing Survey

| **Chairperson** | |
| --- | --- |
| Toshiharu IKAGA * | Keio University |
| **Vice-chairperson** | |
| Yoshihisa FUJINO * | University of Occupational and Environmental Health |
| **Organizer** | |
| Shintaro ANDO * | University of Kitakyushu |
| Tatsuhiko KUBO | Hiroshima University |
| **Committee member** | |
| Wataru UMISHIO | Tokyo Institute of Technology |
| Yuko OGUMA | Keio University |
| Naoki KAGI | Tokyo Institute of Technology |
| Hiroshi KANEGAE | Genki Plaza Medical Center for Health Care |
| Shun KAWAKUBO | Hosei University |
| Yoshinobu SAITO | Kanagawa University of Human Services |
| Keigo SAEKI | Nara Medical University |
| Masaru SUZUKI | Tokyo Dental College Ichikawa General Hospital |
| Tsuyoshi SEIKE * | Tokyo University |
| Takayuki TAJIMA | Tokyo Metropolitan University |
| **Experts committee member** | |
| Maki ITO | Japan Federation of Housing Organizations |
| Hiroshi KOJIMA | Keio University |
| Natsue DOIHARA | Keio University |
| **Adviser** | |
| Takesumi YOSHIMURA * | University of Occupational and Environmental Health |
| Kazuomi KARIO * | Jichi Medical University |
| Tanji HOSHI * | Tokyo Metropolitan University |

*: members of the Research Planning Committee for the Promotion of Smart Wellness Housing

Figure S1. 47 prefectures in 8 regions in Japan

Figure S2. Definition of participants’ exposure temperature

Figure S3. Distribution of average exposure time to living room and bedroom temperature
